# Clinical value of semi-quantitative parameters in ^68^Ga-DOTANOC PET/CT in treatment and diagnostics of cranial meningioma in a single-center retrospective analysis

**DOI:** 10.1186/s41824-024-00204-w

**Published:** 2024-05-15

**Authors:** Friedrich Weitzer, Susanne Stanzel, Elisabeth Plhak, Reingard Maria Aigner

**Affiliations:** Department of Radiology, Division of Nuclear Medicine, Medical University of Graz, Auenbruggerplatz 9A, 8036 Graz, Austria

**Keywords:** ^68^Ga-DOTANOC, Meningioma, Semi-quantitative parameters, PET/CT

## Abstract

**Background:**

The value of somatostatin-analogon PET tracers in theranostics in cranial meningioma has been demonstrated in several studies however, the value of semi-quantitative parameters for therapy and patient outcome is still unclear.

**Methods:**

A retrospective study was performed comparing measured semi-quantitative ^68^Ga-DOTANOC PET/CT parameters (maximum standardized uptake value = SUVmax, mean standardized uptake value = SUVmean, and metabolic tumor volume = MTV) and calculated ratios (SUVmax tumor/to pituitary gland, SUVmax tumor to superior sinus sagittalis), versus WHO grades and overall outcome. Patients with histological confirmed meningioma or high probability for meningioma in previous cranial MRI were eligible.

**Results:**

Thirty-two patients from January 2018 to February 2023 were retrospectively included. WHO grade I meningioma was confirmed in 17 patients, WHO grade II in five patients, WHO grade III in two patients, while in eight patients diagnosis was solely based on MRI and ^68^Ga-DOTANOC PET/CT findings. In 12 cases stable disease was present, in 15 cases radiation therapy was chosen, in three cases neurosurgery was preferred while in two cases palliative care was chosen. Median SUVmax values increased with WHO grade (15.84, 17.22, and 28.4, *p* = 0.134, Kruskal–Wallis-test) and no statistically significant difference was present for MTV, SUVmax, and calculated ratios, although the ratio for SUVmax tumor to superior sinus sagittalis had the lowest value of *p* = 0.067.

**Conclusion:**

Increased SUVmax values in the tumor in ^68^Ga-DOTANOC PET/CT are associated with higher WHO grade, although further studies including larger patient collectives are needed to solidify this hypothesis.

## Introduction

Meningiomas are the most common intracranial tumor, evolving from a mesodermal line. They account for approximately 13–26% of all central nervous tumors (Vernooij et al. 2007; Marosi et al. 2008). Although generally considered benign entities with a slow growth rate, they can show invasive growth and high recurrence rate. Meningiomas are divided into three histological sub-types according to the World Health Organization (WHO) classification: grade I: benign, grade II: atypical, and grade III: anaplastic or malignant. Recurrence rates increase from up to 25% for grade I to up to 94% for grade III. While five-year survival rate for grade I exceeds 80%, it is poorer for malignant and atypical meningiomas at less than 60% (Marosi et al. 2008). Treatment is usually surgical when localized and radiotherapy in more widespread lesions, while peptide receptor radionuclide therapy (PRRT) could achieve stable disease in up to 66.6% (Filippi et al. 2022; Pirisino et al. 2022). Although seen as gold standard in grade III, radiotherapy is debated for grade II and generally not indicated for grade I when resectable (Maggio et al. 2021).

According to the current European Association of Neuro-Oncology (EANO) guideline (Goldbrunner et al. 2021), the diagnosis of intracranial meningioma can be based on MRI and CT scans with high probability in most cases. PET using somatostatin analogs can delineate meningiomas expressing somatostatin receptor 2, although this is not available as standard practice yet. Furthermore, for surgery guidance PET images should be integrated especially for intraosseous meningioma. The most commonly used somatostatin analogs or somatostatin receptor (SSTR) ligands are [^68^Ga-DOTA^0^-Tyr^3^] octreotide (^68^Ga-DOTATOC), [^68^Ga-DOTA^0^-Tyr^3^] octreotide (^68^Ga-DOTATATE), and [^68^Ga-DOTA^0^-^1^NaI^3^] octreotide (^68^Ga-DOTANOC). Gallium-68has a physical half-life of 68min and can be obtained from a Germanium-68/Gallium-68generator system, enabling in-house production without the need for a cyclotron (Galldiks et al. 2017). Since SSTR ligands do not cross the blood/brain barrier, high contrast and excellent target-to-background contrast are provided, enhancing the diagnostic abilities of PET/CT.

## Objective

To determine the value of semi-quantitative parameters in ^68^Ga-DOTANOC PET/CT in intracranial meningiomas in a retrospective single-center study.

## Patients and methods

This retrospective single-center study included patients undergoing ^68^Ga-DOTANOC PET/CT scans at our division from January 2018 to February 2023 for the diagnosis of suspected intracranial meningioma or the restaging of remnant and/or recurrent meningioma. Patients from both sexes and from all ages were eligible.

The Gallium-68labelled DOTANOC ligand was prepared in-house according to the established EANM guideline on current good radiopharmacy practice (cGRPP) (Aerts et al. 2014; Gillings et al. 2022). Gallium-68was obtained from a Germanium-68/Gallium-68Generator (Galli Ad, IRE, Fleurus, Belgium). Quality control was successfully carried out according to the European Pharmacopeia. All prepared products were apyrogenic and sterile with a radiochemical purity > 91%.

All PET/CT examinations were performed on three dedicated PET/CT systems in 3D mode (Discovery MI, GE Healthcare, Milwaukee, WI, U.S.A.; Discovery ST, GE Healthcare, Milwaukee, WI, U.S.A.; and Biograph mCT, Siemens, Erlangen, Germany); patients were unsystematically referred to each scanner. Target dose was 2 MBq kg body weight range 80–200 MBq according to established EANM dose recommendations (Jacobs et al. 2005).

Images of the skull were acquired over 10 min scan time 60 min after i.v. injection while additional images of the body-stem from skull base to mid-thighs were acquired in selected cases due to suspicion of ^68^Ga-DOTANOC avid extracranial malignancy by discontinuously craniocaudal bed movement and an acquisition time of 2 min per bed position afterwards regardless of BMI values. Transmission CT scans for attenuation correction were acquired using helical mode without the use of a contrast agent. Both PET and CT scans were reconstructed with a slice thickness of 3.75 mm. All studies were interpreted by two experienced Nuclear Medicine physicians in consensus reading.

^68^Ga-DOTANOC positive tumor lesions were visually identified as focal uptake significantly higher than adjacent background activity, not associated with physiological uptake in the pituitary gland as recommend by the generally accepted international EANM/SNMMI criteria (Bozkurt et al. 2017). Volumes of interest (VOI) to acquire SUVmax, SUVmean and MTV were manually drawn over pathological uptake in the tumor, avoiding the very high physiological activity in the pituitary gland. VOIs were also drawn to acquire SUVmax values in the pituitary gland (SUVpit) and superior sinus sagittalis (SUVsss) to obtain raw data for calculating the SUVmax ratio tumor/pituitary gland (SUVRpit) and SUVmax ratio tumor/superior sinus sagittalis (SUVRsss).

Image analysis and semi-quantitative data for both primary tumor and metastasis were acquired on the manufacturer supplied workstation (AW Server 3.2 Ext. 4.0, GE Healthcare, Milwaukee, WI, U.S.A.) using the manufacturer provided auto-snake tool with automatic threshold definition pre-set to 42% of SUVmax. The Kruskal–Wallis-test was used to compare SUVmax, SUVmean, SUVRpit, SUVRsss, and MTV between WHO grades in meningioma. Statistical examination was performed with IBM™ SPSS™ Statistics version 29.0 (Chicago, Ill, U.S.A.).

## Results

From January 2018 to February 2023, 1309 ^68^Ga-DOTANOC PET/CT examinations were performed at our division. The absolute and relative number of examinations increased over time. In 32/1309 selected cases (2.44%, 26 female) intracranial meningioma was already known or highly suspected. (See Table 1 for patient characteristics and demographics).

**Table 1:** Patient characteristics including previous treatment, WHO histological classification of the intracranial meningioma

Analyzed parameters	Patients	Percentage
Female (%)	26/32	81.3%
*Age (years)*		
Mean	54.94	Range: 6–82 years
Median	58.34	
Standard deviation	15.38	
*Body mass index*		
Mean	26.51	Range: 17.8–40.4
Median	25.08	
Standard deviation	5.71	
*Previous treatment*		
Neurosurgery	17	45.9%
Neurosurgery and radiation therapy	7	18.9%
Radiation therapy	6	16.2%
None	7	18.9%
*WHO histological classification*		
Grade I	17	45.9%
Grade II	6	16.2%
Grade III	2	5.4%
No histology present	12	32.4%
MRI presumptive diagnosis only	12	32.4%

**Fig. 1:**
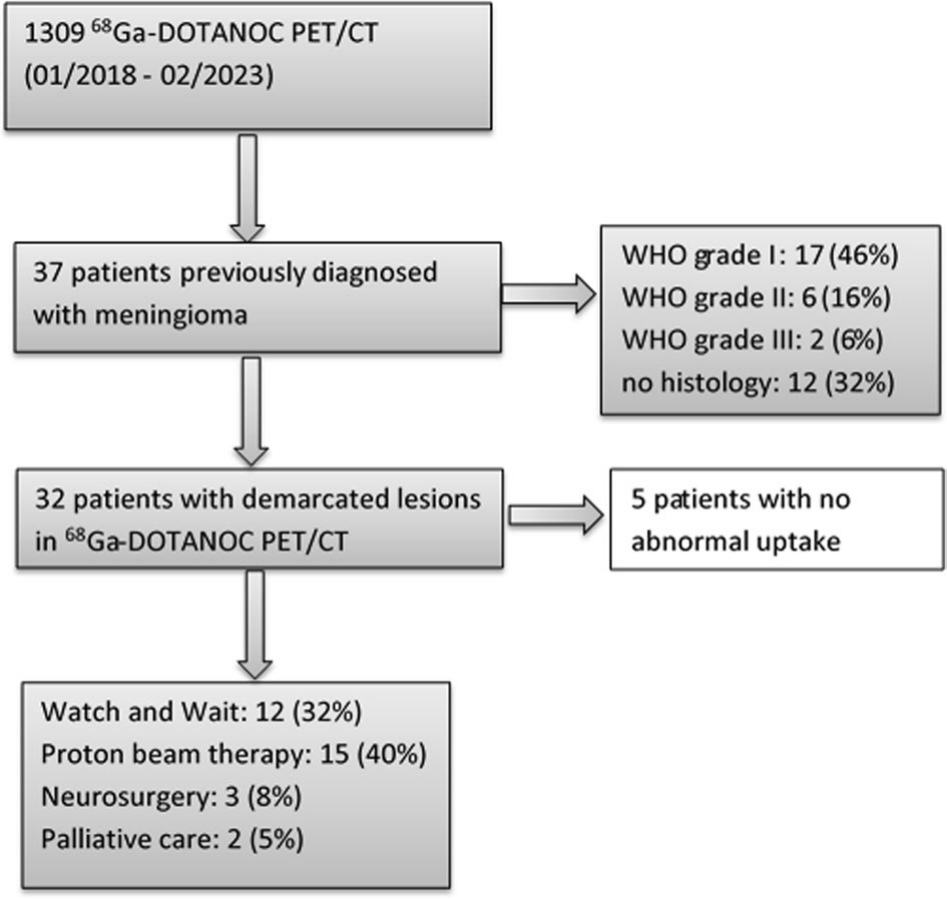
Flow-chart of the included patients and therapy decisions based on PET/CT

Previous treatment was performed in 30 patients (81.1%), including neurosurgery and /or radiation therapy, while histologically proven meningioma was available in 25 patients (67.6%). Regarding the WHO histological classification WHO grade I was present in 17 cases (45.9%), WHO grade II in 6 cases (16.2%) and WHO grade III in only 2 cases (5.4%). In 12 cases (32.4%) stable disease was present, in 15 cases (40.5%) proton beam radiation therapy was chosen, in three cases (8.1%) neurosurgery was preferred, while in two cases (5.4%) palliative care was chosen due to multiple recurrent intracranial lesions. All depicted meningiomas showed a significant ^68^Ga-DOTANOC uptake with a median SUVmax value of 17.3 (range: 6.12–64.78) a median SUVmean of 8.4 (range: 1.78–24.06) and a median MTV of 4.89cm^3^ (range: 0.16–76.4 cm^3^) (See Fig. 1 for graphic display).

**Fig. 2:**
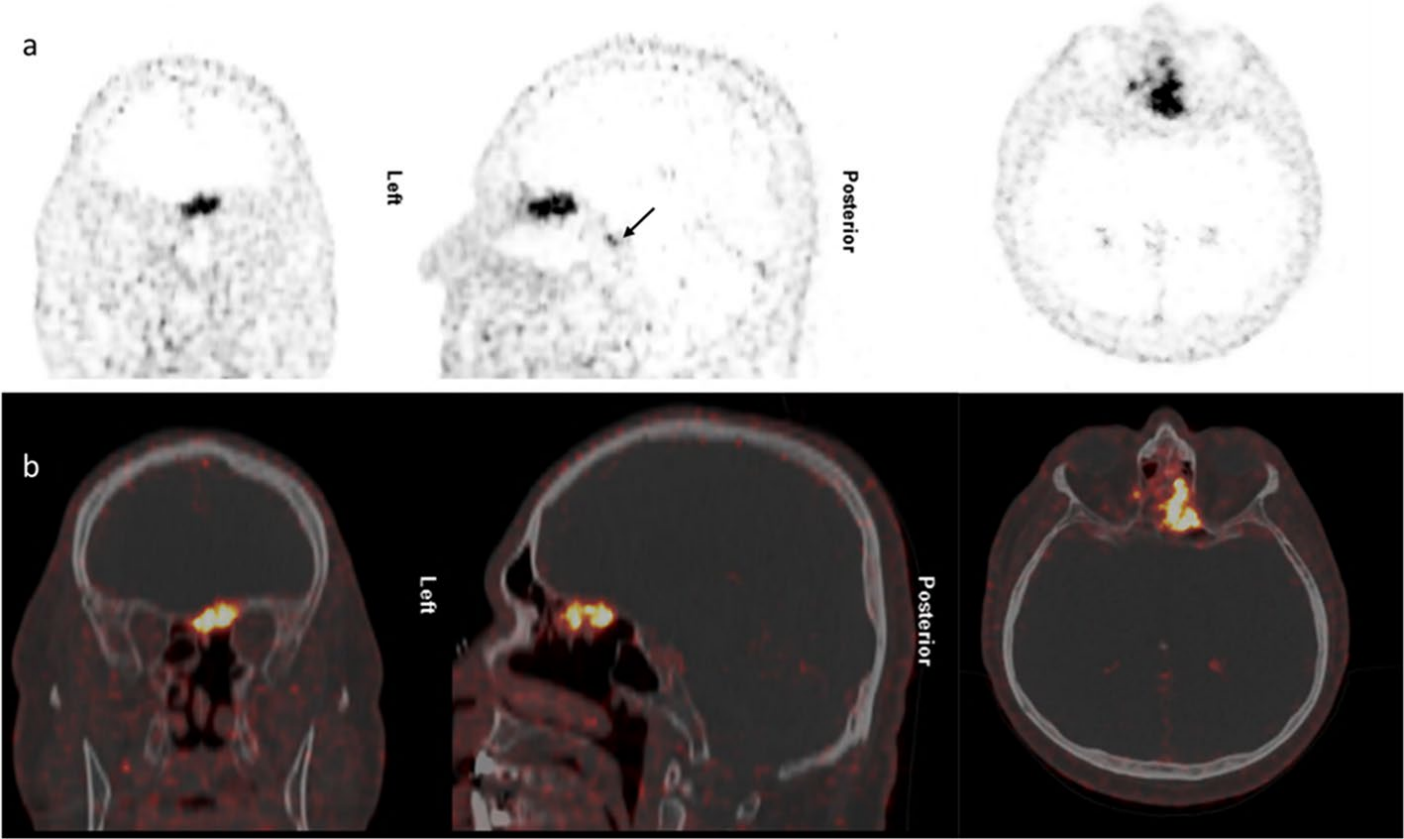
^68^Ga-DOTANOC PET/CT showing meningioma of the left olfactory nerve with intra- and extracranial tumor masses. Patient was a 64-year-old male neurosurgical treated before PET/CT. Stable disease and WHO grade II allowed a “watch and wait” strategy. Arrow: Physiological tracer uptake in the pituitary gland **a** PET images, **b** fused images in three planes

**Fig. 3:**
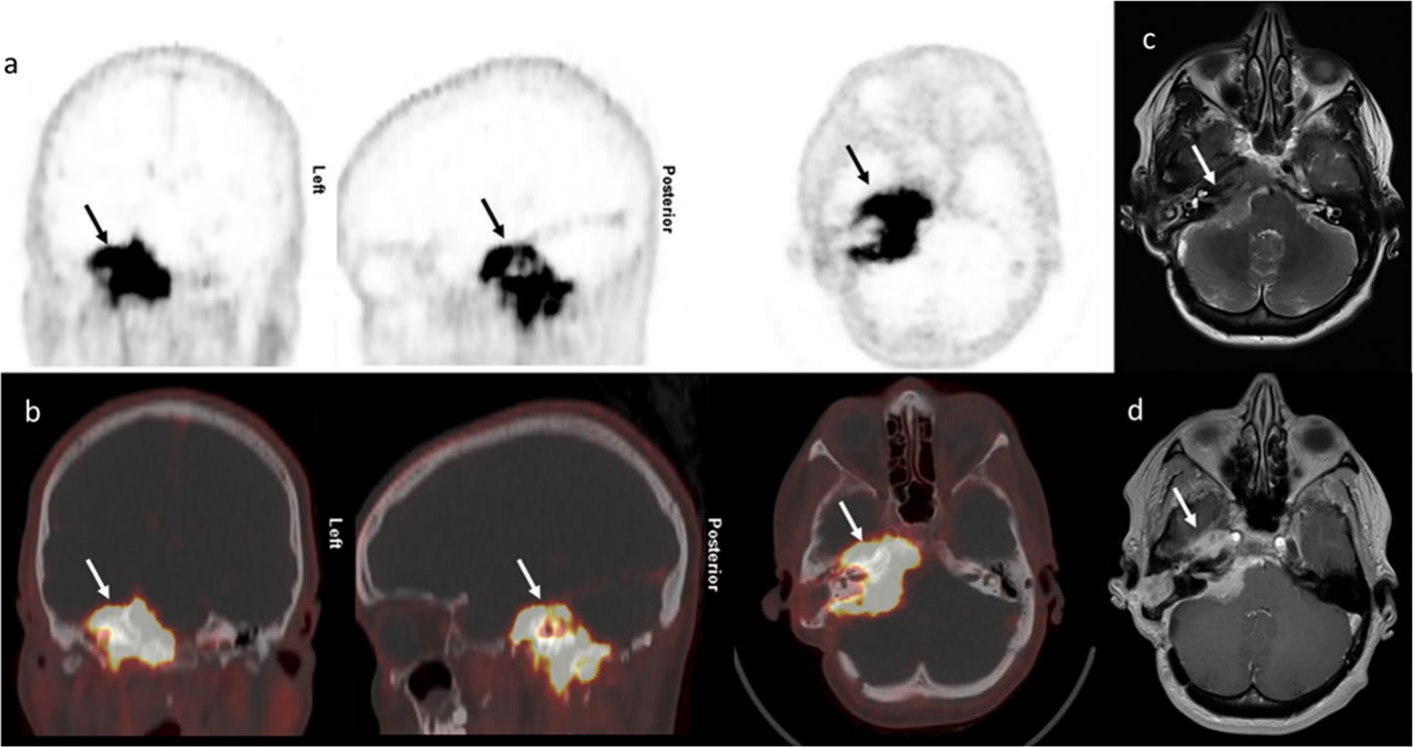
^68^Ga-DOTANOC PET/CT showing meningioma of the right alisphenoidal and petrous bone in a 47-year-old female patient with resected WHO grade I meningioma (arrow). Due to the large tumor masses extended radiation therapy was scheduled. **a** PET images, **b** fused images in three planes, **c** transversal T2 MRI image, **d** transversal contrast-enhanced T1 MRI image

**Fig. 4:**
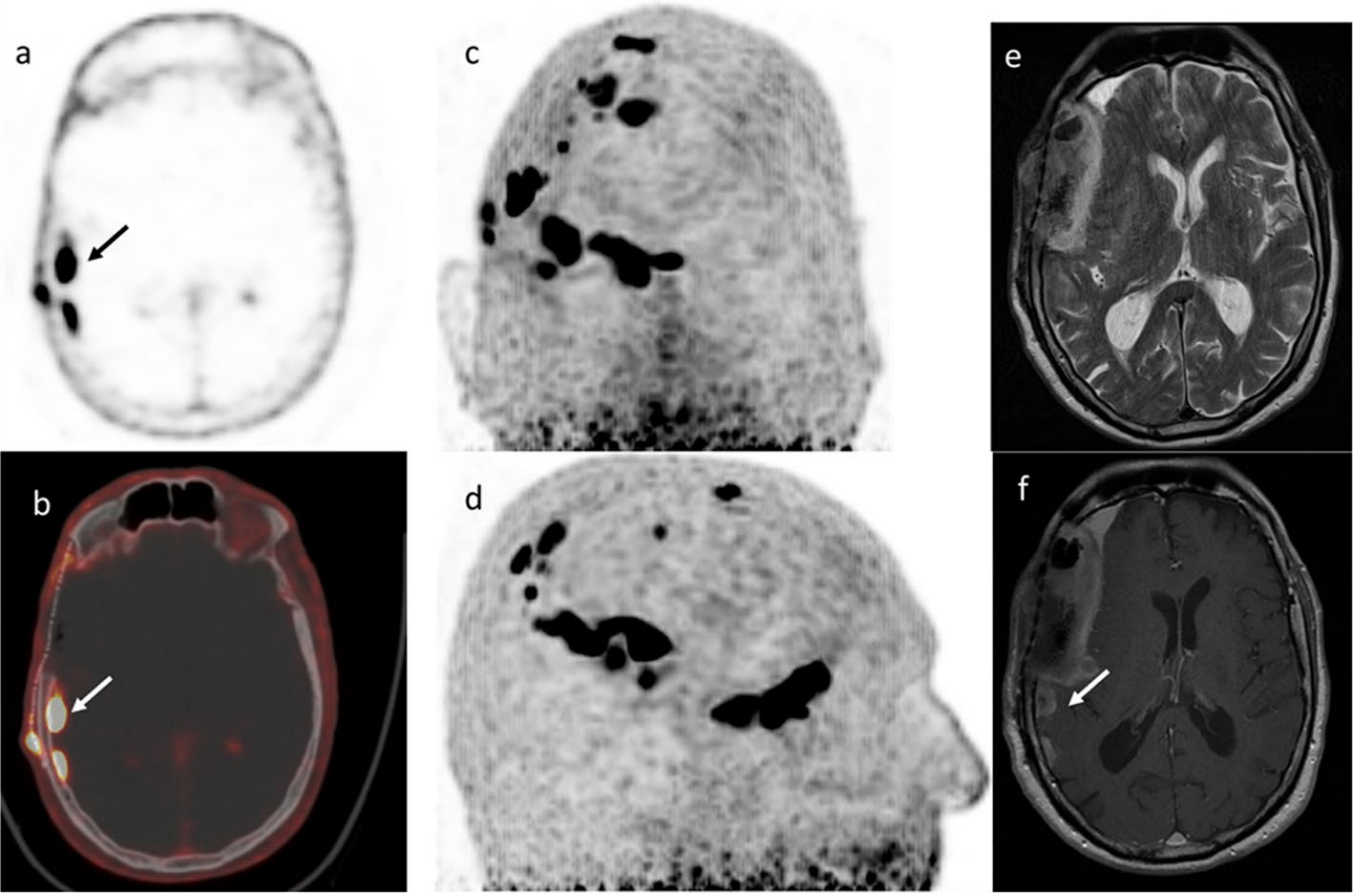
WHO grade III meningioma in a 74-year-old female patient with multiple operations. Arrow shows two meningioma next to the post-surgical bone defect. Maximum intensity projection (MIP) mode in panel c and d shows multiple meningeal lesions. Due to the widespread disease and the elevated age of the patient palliative care was chosen. **a** transversal PET image, **b** transversal fused PET/CT image, **c** anterior view of MIP mode, **d** right lateral view of MIP mode, **e** transversal MRI T2 image, **f** transversal contrast-enhanced MRI T1 image

Significant ^68^Ga-DOTANOC uptake with a good contrast to the surrounding tissue and a sharply demarcated tumor border was present in all meningiomas, confirming the value of ^68^Ga-DOTANOC PET/CT in the diagnosis of meningioma. No significant differences between visual appearance of tumor volume and extension and semi-automated acquisition of MTV were observed (See Figs. 2, 3, and 4 for a representative case). In protocols with additional scans from skull base to mid-thighs no pathological extracranial uptake was present.

Analysis of semi-quantitative parameters, the main objective of this study, showed an increase of median SUVmax in the tumor associated with WHO grade while no association was present for median MTV (15.84 in WHO grade I, 17.22 in WHO grade II, and 28.4 in WHO grade III and 5.26, 3.23, 18.59, respectively), however no significance could be demonstrated (*p* = 0.134 for SUVmax and *p* = 0.216 for MTV). No statistical significance was present between ^68^Ga-DOTANOC uptake using SUVmean values in the tumor and WHO grade (*p* = 0.180), and for calculated tumor to superior sinus sagittalis ratio (median 6.24, 4.34, and 16.47; *p* = 0.067, while the tumor to pituitary gland ratio did not differ (0.98, 1.10, 0.95; *p* = 0.405) (See Table 2).

**Table 2:** Semi-quantitative parameters for different WHO grades

n = 25	SUVmax	SUVmean	MTV	SUVpit	SUVsss	SUVRpit	SUVRsss
*WHO grade I (n* = *17)*							
Mean	16.45	8.35	11.74	15.76	2.36	1.09	7.36
Median	15.84	8.53	5.26	14.53	2.39	0.98	6.24
Standard deviation	7.69	4.05	17.25	5.60	0.58	0.42	3.75
Minimum	6.12	1.78	1.42	6.27	1.16	0.38	2.51
Maximum	38.68	19.96	76.04	27.14	3.18	2.03	16.15
*WHO grade II (n* = *6)*							
Mean	18.57	8.00	6.76	13.21	3.55	1.87	5.97
Median	17.22	7.78	3.23	15.19	4.33	1.10	4.34
Standard deviation	5.13	1.49	5.69	5.19	1.21	1.51	2.87
Minimum	11.60	6.25	1.92	5.57	1.77	0.95	3.79
Maximum	27.18	10.02	16.45	18.45	4.69	4.88	11.33
*WHO grade III (n* = *2)*							
Mean	28.42	10.44	18.59	29.87	1.71	0.95	16.47
Median	28.42	10.44	18.59	29.87	1.71	0.95	16.47
Standard deviation	5.29	0.19	4.54	1.28	0.15	0.14	1.65
Minimum	23.13	10.25	14.04	28.59	1.56	0.81	14.83
Maximum	33.70	10.63	23.13	31.14	1.86	1.08	18.12

*SUVmax* maximum standard uptake value in the tumor, *SUVmean* mean standard uptake value in the tumor, *MTV* metabolic tumor volume, *SUVpit* maximum standard uptake value in the pituitary gland, *SUVsss* maximum standard uptake value in the superior sinus sagittalis, *SUVRpit* tumor to pituitary gland ratio, *SUVRsss* tumor to sinus sagittalis ratio

## Discussion

Diagnosis and determination of topographic extension of intracranial tumors is based on neuroimaging consisting mainly MRI and seldom cranial CT with or without contrast agent. However, these radiological modalities have limitations, especially when posttherapeutic changes are present (Galldiks et al. 2017). ^68^Ga-DOTANOC PET/CT has the potential to present a meaningful alternative if previous MRI findings are unclear as proven in a recently published systematic review (Filippi et al. 2022). Meningioma unlike other intracranial tumors has a high incidence of somatostatin receptors posing a potential target for both diagnosis and therapy (Dutour et al. 1998). Furthermore, a retrospective study by Milosevic et al. (Milosevic et al. 2023) showed no diagnostic improvement for detection and differentiation of meningioma using PET/MRI and a dedicated MRI protocol.

Before discussing the results concerning the meningiomas it is noteworthy that a higher variability between the physiological tracer uptakes in the pituitary gland with a SUVpit (SUVmax values) ranging from minimum 5.57 to maximum 52.52 (median 18.19) than in the superior sinus sagittalis with a SUVsss (SUVmax values) ranging from minimum 0.94 to maximum 4.69 (median 2.37) was found. This finding is consistent to findings reported by Campos Neto et al. (2022) reporting a range from 12.75 to 32.03.

A similar approach to this topic was performed by Kim et al. (2022) evaluating semi-quantitative parameters in ^68^Ga-DOTATATE PET/MRI in 166 meningiomas. Interestingly, in this study the SUVmax values showed the highest sensitivity, while the SUVRsss values showed the highest specificity. The lack of reproducibility in our study may be caused by the smaller and possibly more heterogeneous patient collective.

Current guidelines including the 2017 EANM guideline for PET/CT imaging of neuroendocrine neoplasms (Bozkurt et al. 2017) mention the usability of radiolabeled somatostatin analogues for the diagnosis of meningioma without going into detail or describing the value of semi-quantitative parameters. No further amendments concerning this topic were made in a 2023 preprint SNMMI procedure standard/EANM practice guideline for SSTR receptor PET (https://www.eanm.org/publications/guidelines/oncology/). A different and more concise approach is stated in the current EANO guideline (Goldbrunner et al. 2021) on meningioma were the value of somatostatin receptor PET for distinguishing tumor from healthy tissue and postoperative tissue changes is mentioned, however PET/CT is still not seen as standard practice due to the limited availability. Despite the recent advances in hybrid imaging and the widespread use of CT for attenuation correction there are still pitfalls and limitation due to patient movement during the examination. Radiation exposure poses an additional limiting factor especially in children (Salmon et al. 2015).

The manufacturer supplied automatic set threshold of 42% of SUVmax proved adequate for tumor volume determination and MTV which is contradictory to the findings described by Kriwanek et al. (Kriwanek et al. 2022) examining the inter-observer variability of target delineation of meningioma were a threshold of 14% of SUVmax was deemed adequate. In the same study, a general increase in delineated tumor volume was present for most physicians, although a major drop was seen in one physician.

This small retrospective study has several limitations: First, the number of patients is limited, so a larger patient collective could produce more stable results. Secondly, a prospective setting comparing the same patient collective, and outcome would produce more valid data, however this would be more time consuming especially regarding the individual patient outcome.

## Conclusion

^68^Ga-DOTANOC PET/CT plays a key-role in the diagnosis and determination of the topographic extension in intracranial meningioma. Significant ^68^Ga-DOTANOC uptake with a good contrast to the surrounding tissue and a sharply demarcated tumor border was present in all meningiomas. Increased SUVmax and MTV values in the tumor in ^68^Ga-DOTANOC PET/CT are associated with higher WHO grade. No correlation was present for calculated tumor to pituitary gland and tumor to superior sinus sagittalis ratios, although the ratio for SUVmax tumor to superior sinus sagittalis had the lowest value of *p* = 0.067. Nevertheless, further studies including larger patient collectives are needed to solidify this hypothesis.

## Data Availability

The datasets analyzed during the current study are available from the corresponding author on reasonable request.
